# Lipids assist the membrane insertion of a BAM-independent outer membrane protein

**DOI:** 10.1038/srep15068

**Published:** 2015-10-14

**Authors:** Gerard H. M. Huysmans, Ingrid Guilvout, Mohamed Chami, Nicholas N. Nickerson, Anthony P. Pugsley

**Affiliations:** 1 Institut Pasteur, Molecular Genetics Unit, Departments of Microbiology and of Structural Biology and Chemistry, rue du Dr. Roux, 75724 Paris Cedex 15, France, CNRS ERL3526, rue du Dr. Roux, 75724 Paris Cedex 15, France; 2 Institut Pasteur, Laboratory of Macromolecular Structure and Signalling, Departments of Microbiology and of Structural Biology and Chemistry, rue du Dr. Roux, 75724 Paris Cedex 15, France; 3 C-CINA Center for Cellular Imaging and NanoAnalytics, Biozentrum, University of Basel, CH 4058 Basel, Switzerland

## Abstract

Like several other large, multimeric bacterial outer membrane proteins (OMPs), the assembly of the *Klebsiella oxytoca* OMP PulD does not rely on the universally conserved β-barrel assembly machinery (BAM) that catalyses outer membrane insertion. The only other factor known to interact with PulD prior to or during outer membrane targeting and assembly is the cognate chaperone PulS. Here, *in vitro* translation-transcription coupled PulD folding demonstrated that PulS does not act during the membrane insertion of PulD, and engineered *in vivo* site-specific cross-linking between PulD and PulS showed that PulS binding does not prevent membrane insertion. *In vitro* folding kinetics revealed that PulD is atypical compared to BAM-dependent OMPs by inserting more rapidly into membranes containing *E. coli* phospholipids than into membranes containing lecithin. PulD folding was fast in *di*C_14:0_-phosphatidylethanolamine liposomes but not *di*C_14:0_-phosphatidylglycerol liposomes, and in *di*C_18:1_-phosphatidylcholine liposomes but not in *di*C_14:1_-phosphatidylcholine liposomes. These results suggest that PulD efficiently exploits the membrane composition to complete final steps in insertion and explain how PulD can assemble independently of any protein-assembly machinery. Lipid-assisted assembly in this manner might apply to other large OMPs whose assembly is BAM-independent.

The outer membrane of Gram-negative bacteria contains many proteins with diverse functions that, besides making the membrane semi-permeable to nutrients and solutes, are critical to cellular organisation, fitness and survival^1^. It is therefore crucial for the bacterium to ensure that the outer membrane contains the right amount of functional proteins. Once passaged through the Sec-translocon, outer membrane proteins (OMPs) rely on one or several periplasmic proteins to chaperone them to the outer membrane^2^. Many OMPs have a β-sheet transmembrane topology and are passed on from these chaperones to the outer membrane embedded β-barrel assembly machinery (BAM) that catalyses their membrane insertion^3^. Although the insertion of proteins into the inner membrane is linked to the hydrolysis of ATP and the proton-motive force^4^, such classical energy sources are not available to catalyse protein insertion into the outer membrane. BAM is proposed to help overcome the energy barrier required for OMP insertion in the presence of lipids with phosphatidylethanolamine (PE) headgroups^5^,^6^,^7^.

Unlike the pathway followed by most OMPs, the assembly of some, but not all^8^, large α-helical and β-sheet secretion pores is BAM-independent^9^,^10^. Membrane insertion of BAM-dependent OMPs might occur through a process of β-strand augmentation during which BAM forms a pore-chimera with the growing substrate OMPs^11^,^12^. How large BAM-independent OMP oligomers might insert into the membrane remains unclear. However, their assembly might rely on a different membrane insertion mechanism that, regardless of the transmembrane secondary structure, prevents the formation of a large open channel in the membrane. Here we address this question using the secretin PulD as a model system.

Secretins are a large and important class of outer membrane proteins that form multimeric exit portals of secretion systems for enzymes, virulence factors, surface pili and filamentous phages^13^. PulD from the type II secretion system from *Klebsiella oxytoca*^14^ is a prototype of the secretin family. In *K. oxytoca*, and when expressed in its entirety in *E. coli*, this system secretes the enzyme pullulanase (PulA)^14^. PulD consists of a modular periplasmic N-domain containing four subdomains (named N_0_-N_3_)^15^,^16^, a membrane embedded C-domain that is conserved throughout the secretin family^15^,^17^, and a C-terminal S-domain that interacts with a PulD-dedicated chaperone, the lipoprotein PulS^18^,^19^. PulD targeting to the outer membrane occurs *via* the Lol-pathway and is strictly PulS-dependent^20^. In the absence of PulS, PulD inserts into the inner membrane and induces a stress response that includes massive production of the protein PspA^21^,^22^. Whereas the nature of the PulD transmembrane topology remains to be determined, its BAM-independence for outer membrane assembly is well-established^23^.

Many OMPs, including PulD, can fold spontaneously *in vitro* in the presence of liposomes^24^,^25^, providing a method to dissect the roles of chaperones during the folding of these proteins in a controlled *in vitro* environment^5^,^7^,^26^,^27^,^28^. An equivalent approach has not yet been used for PulS in PulD assembly. To address whether PulS has additional roles besides outer membrane targeting and how PulD overcomes the energetic barrier for efficient assembly, we took advantage of the spontaneous *in vitro* folding of PulD in a coupled transcription-translation reaction containing liposomes. *In vitro*, PulD folding achieves optimal efficiency when only a short sequence of the N-terminus and the N_3_-subdomain precede the C- and S-domains^25^. Truncation of N_0_-N_2_ does not affect *in vivo* assembly, indicating that all *in vivo* interactions required for correct assembly are present in this truncated PulD variant^25^. We previously showed that this truncated secretin, PulD^28–42/259–660^, folds *via* a multistep mechanism: membrane adsorbed monomers dodecamerise into a prepore that then inserts into the membrane^29^. Secretins produced in this manner are indistinguishable from secretins purified from native membranes according to their secondary and quaternary structure and their biochemical properties. Here, we report the effects of adding PulS to the *in vitro* PulD synthesis reaction and of cross-linking PulS to PulD *in vivo* on the acquisition of native state determinants. We further examine the effects of changes in the membrane composition to *in vitro* folding kinetics and propose a folding model in which membrane lipid properties directly influence membrane insertion without the assistance of proteinaceous co-factors. In what follows, we use the terms ‘folding’ and ‘assembly’ to distinguish between the *in vitro* and the *in vivo* processes, respectively.

## Results

### sPulS facilitates rapid PulD^28–42/259–660^ multimerisation in lecithin liposomes

We previously observed that PulD^28–42/259–660^ multimerisation *in vitro* is inversely dependent on the concentration of lecithin in the coupled synthesis and insertion reaction^29^. To find conditions under which the effects of adding PulS to the *in vitro* coupled transcription-translation reaction could be measured, we added a non-lipidated form of PulS (sPulS) to the reaction mixture in the presence of increasing amounts of lecithin before commencing PulD^28–42/259–660^ synthesis. Although the overall production of PulD^28–42/259–660^ is lower at lecithin concentrations above 27 mM, we have shown previously that this does not impair the analysis of initial PulD multimerisation^29^. sPulS was used because the presence of a lipid anchor would (1) require the use of detergent that interferes with liposome integrity and (2) physically restrain PulS on the lipid surface rather than being free in solution to recruit PulD monomers. We established previously that PulS produced in this way interacts efficiently with PulD^19^,^30^,^31^.

**Figure 1:**
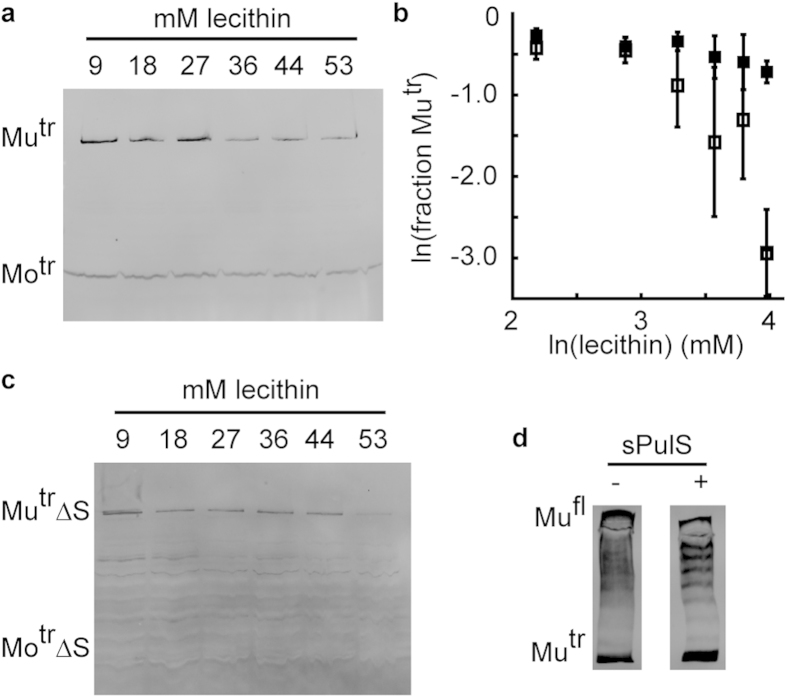
Effect of sPulS on PulD folding. (**a**) Initial multimerisation of PulD^28–42/259–660^ in the presence of 0.2 μg/μl sPulS and increasing quantities of lecithin (as indicated) after 6 min of PulD-synthesis. (**b**) Double logarithmic plot of the initial multimerisation of PulD^28–42/259–660^ (◼) in (**a**). Errors represent S.D. over 3 independent measurements. Initial PulD multimerisation in the absence of sPulS is also shown (◻) (from ref. 29). (**c**) Initial multimerisation of PulDΔS^28–42/259–598^ in the presence of 0.2 μg/μl sPulS and increasing quantities of lecithin (as indicated). PulDΔS^28–42/259–598^ degradation results in multiple bands below the multimer (Mu^tr^ΔS); the approximate position of the monomer is indicated (Mo^tr^ΔS). (**d**) Mixed multimer formation between PulD^28–42/259–660^ and and PulD^fl^ in equimolar ratios in the presence of 53 mM lecithin and 0.2 μg/μl PulS as indicated. Mu^tr^ and Mo^tr^ indicate the migration position of multimeric and monomeric PulD^28–42/259–660^ species, respectively. Mu^fl^ indicates the position of full-length PulD.

At low lecithin concentrations, initial PulD^28–42/259–660^ multimerisation is too fast to measure a contribution of sPulS (Fig. 1a and b). However, at 53 mM lecithin, initial PulD^28–42/259–660^ multimerisation was markedly lower in the absence of sPulS (Fig. 1b, open squares) than in its presence (Fig. 1b, filled squares). A PulD variant lacking the S-domain, PulDΔS^28–42/259–598^, is unable to interact stably with PulS^18^,^32^. PulDΔS^28–42/259–598^ synthesis would therefore be expected to show a large inverse dependence on the lecithin concentration at high concentrations even in the presence of sPulS. Rapid degradation of PulDΔS^28–42/259–598^ and its poor recognition by the anti-PulD antibody prevented in depth analysis of PulDΔS^28–42/259–598^ multimerisation. Nonetheless, immunoblots clearly show that initial PulDΔS^28–42/259–598^ multimerisation was very low at 53 mM lecithin with sPulS present in the reaction mixture before synthesis (Fig. 1c). This result thus demonstrates that the sPulS-mediated increased initial multimerisation of PulD^28–42/259–660^ was specific to the binding of the S-domain of PulD^28–42/259–660^ to sPulS.

The observed increase in initial PulD^28–42/259–660^ multimerisation in the presence of sPulS could stem from a direct kinetic advantage by rapid association of the PulD^28–42/259–660^/sPulS complex with the lecithin surface, from a PulS-induced conformational advantage for PulD^28–42/259–660^ oligomerisation, or from a change in the PulD^28–42/259–660^ folding mechanism. If sPulS binding induces the formation of small oligomers prior to dodecamerisation, then the membrane dependent multimerisation reaction would reduce in order, lowering its inverse dependence on the lecithin concentration: dodecamerisation by monomeric addition results in a dependence of the initial multimerisation of up to twelve, hexamerisation of dimers in a dependence of up to six and so forth. Such oligomers can be observed by creating mixed multimers between full-length PulD (PulD^fl^) and PulD^28–42/259–660^, which separate as a regular 13-step ladder by SDS-polyacrylamide electrophoresis^29^. If sPulS helps form oligomers that are obligatory intermediates in dodecameristion, then distinct steps should disappear when PulD^fl^ and PulD^28–42/259–660^ synthesised separately in the presence of sPulS are mixed post-synthesis^29^. However, PulD^28–42/259–660^ formed regular ladders with PulD^fl^ in the presence or absence of sPulS (Fig. 1d), making it unlikely that sPulS induces the formation of small obligate oligomers prior to dodecamerisation.

### sPulS does not accelerate other PulD^28–42/259–660^ folding steps in lecithin liposomes

**Figure 2:**
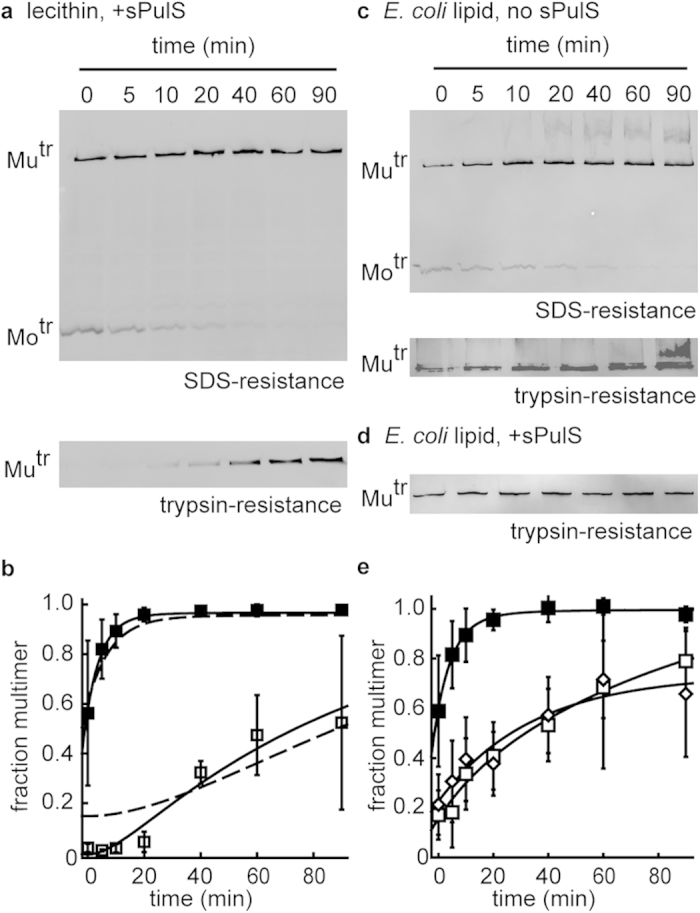
Kinetics of PulD^28–42/259–660^ folding in lecithin or *E. coli* lipid liposomes in the presence and absence of sPulS. (**a**) Kinetics of PulD^28–42/259–660^ folding after 10 min synthesis in the presence of 0.2 μg/μl sPulS and 53 mM lecithin, measured by the acquisition of SDS- and trypsin-resistance of the PulD^28–42/259–660^ multimer. (**b**) Plot of the multimerisation kinetics measured by SDS-resistance (◼) and by trypsin-resistance (◻) in (**a**) following band quantification by densitometry. Errors represent S.D. over 3 independent measurements. Dotted lines represent the fits of the PulD^28–42/259–660^ multimerisation kinetics in the absence of PulS (from ref. 29). (**c**) Kinetics of PulD^28–42/259–660^ folding in the presence of 53 mM *E. coli* polar extract liposomes measured by the acquisition of SDS- and trypsin-resistance of the PulD^28–42/259–660^ multimer and measured by the acquisition of trypsin-resistance in the presence of 0.2 μg/μl sPulS (**d**). (**e**) Plot of the multimerisation kinetics measured by SDS-resistance (◼) and by trypsin-resistance in the absence (◻) and presence of (◊) sPulS in (**c,d**) following band quantification by densitometry. Errors represent S.D. over 3 independent measurements. Mu^tr^ and Mo^tr^ indicate the migration position of multimeric and monomeric PulD^28–42/259–660^ species, respectively. Only multimers are shown for the trypsin resistant state, as monomers were completely digested.

Since sPulS caused more rapid initial multimerisation of PulD^28–42/259–660^ at 53 mM lecithin, it was next investigated whether the effect of PulS extended to subsequent kinetic steps in PulD^28–42/259–660^ folding. PulD^28–42/259–660^ folding under these conditions was monitored by SDS-treatment and by subjecting PulD^28–42/259–660^ to trypsinolysis at increasing time points after 10 min synthesis (Fig. 2a). Despite the more rapid PulD^28–42/259–660^ multimerisation in the presence of sPulS immediately after 6 min synthesis, sPulS did not significantly accelerate other phases in PulD^28–42/259–660^ folding (Fig. 2b). Previously, PulD^28–42/259–660^ folding was characterised by two rate constants in a multistep sequential process with simultaneous acquisition of SDS- and urea-resistance, followed by trypsin-resistance of the native protein core upon membrane insertion^29^. Here, data scattering warranted data fitting to a single exponential equation only. The rate constant of 0.18 ± 0.01 min^−1^ agreed well with the fast rate constant of 0.14 ± 0.04 min^−1^ reported in the absence of sPulS^29^. As was the case in the absence of sPulS, trypsin-resistance in lecithin liposomes was acquired after a delay of approximately 20 min (Fig. 2b). Together, the data demonstrate that sPulS only improves the efficiency of initial steps in PulD^28–42/259–660^ folding in lecithin liposomes *in vitro*.

### E. coli lipids do not enhance sPulS dependent PulD^28–42/259–660^ folding

Lipids with phosphatidylcholine (PC)-headgroups (like lecithin) have proven successful for the *in vitro* folding of many OMPs from their chemically denatured state^24^, but bacterial membranes rarely contain PC-headgroups^33^,^34^. The inner leaflet of bacterial outer membranes consists mostly of lipids with phosphatidylethanolamine (PE)-headgroups (up to 90 %) and phosphatidylglycerol (PG)-headgroups^34^. The outer leaflet is exclusively composed of lipopolysaccharides^1^. Most chemically denatured bacterial OMPs fold only inefficiently *in vitro* in membranes derived from native sources like *E. coli*^24^. However, PulD^28–42/259–660^, multimerised efficiently after 10 min synthesis in the presence of *E. coli* liposomes prepared from *E. coli* polar extract lipids (Fig. 2c). The absence of a delay in the acquisition of trypsin-resistance indicated that PulD^28–42/259–660^ acquired its final native state efficiently and faster in the presence of *E. coli* lipids than in the presence of lecithin liposomes (Fig. 2b,c,e).

To investigate whether sPulS could further accelerate PulD^28–42/259–660^ membrane insertion into *E. coli* membranes, the effect of adding sPulS prior to PulD^28–42/259–660^ synthesis on the acquisition of PulD^28–42/259–660^ trypsin-resistance was measured. As in lecithin liposomes, the addition of sPulS to the synthesis reaction did not increase the rate of PulD^28–42/259–660^ folding into its trypsin-resistant native state in the presence of *E. coli* lipids (Fig. 2d,e). Thus, whereas PulD^28–42/259–660^ membrane insertion is rapid in the presence of liposomes prepared from *E. coli* lipids, the addition of sPulS did not reveal an additional kinetic advantage in the presence of these lipids. We cannot exclude that the PulS lipid-anchor plays an additional role in the PulD folding process, for example by creating membrane defects or enforcing a particular orientation with respect to the membrane.

### PulS attachment does not prevent PulD assembly *in vivo*

**Figure 3:**
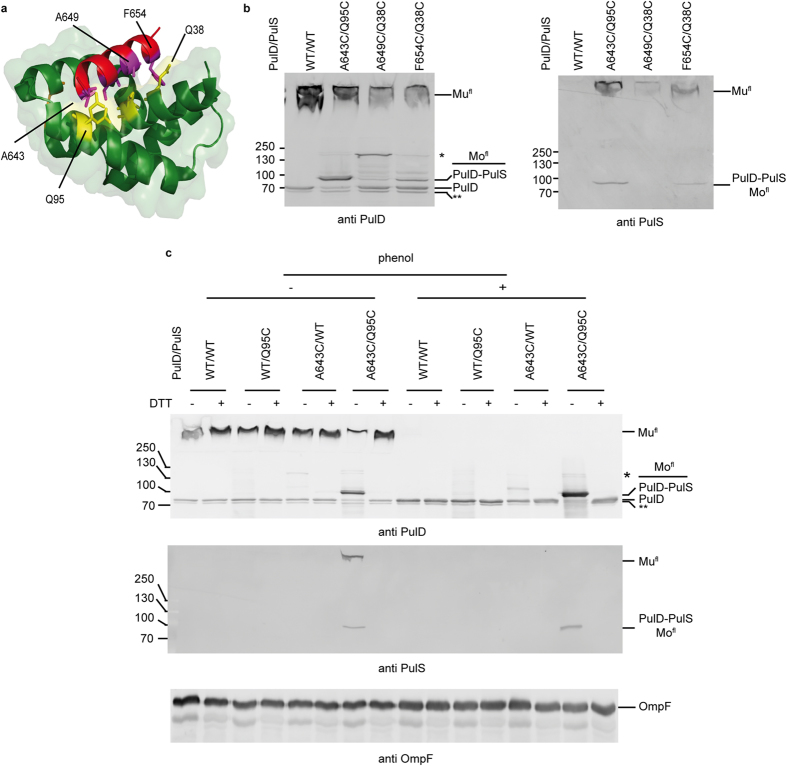
Cross-linking efficiency of cysteine bridges between PulD^fl^ and PulS *in vivo*. (**a**) Cartoon representation of *K. oxytoca* PulS (green) containing the binding peptide of PulD (red) based on the OutD-OutS complex (PDB 4K0U). Residues lining the bottom of the binding cleft in PulS and interacting residues on PulD^fl^ are shown as sticks in yellow and magenta, respectively, and those substituted into cysteines are labelled. Two side-chain orientations are shown for Q95, as in the deposited PulS structure (PDB 4A56). (**b**) Cross-linking efficiency between PulD^fl^ and PulS variants, as indicated. (**c**) Specificity of the PulD^fl^_A643C_-PulS_Q95C_ cross-link. The cysteine cross-link was reduced upon DTT treatment and multimers dissociated by phenol extraction, as indicated. OmpF was used as a loading control before and after phenol extraction. In (**b,c**) immunoblots after SDS-PAGE of total cell extracts were stained with anti PulD, anti PulS and anti OmpF antibodies as indicated. Numbers on the left side of blots in (**b**) and (**c**) indicate migration positions of the molecular weight markers in kDa. Mu^fl^ and Mo^fl^ indicate the migration position of multimeric and monomeric PulD^fl^ species, respectively. * Indicates likely cross-linked dimers between two PulD^fl^ monomers with cysteines; ** Indicates trimmed PulD^fl^ monomers that escape outer membrane targeting^20^.

The lack of any effect on late steps in PulD^28–42/259–660^ folding kinetics might reflect that the PulD^28–42/259–660^/sPulS complex dissociates once it adsorbs onto the lipid surface or forms a dodecamer. Complex dissociation could be a prerequisite for the formation of a native, secretion-competent PulD complex in the outer membrane. To examine further whether this is the case, a series of cysteine variants in PulD^fl^ and PulS was created that would cross-link spontaneously upon interaction *in vivo*. Their capacity to form an efficient cross-linked PulD^fl^-PulS product, to secrete PulA and to induce a PspA-response was examined. The choice of residues for substitution by cysteines was based upon the crystal structures of PulS^31^ and that of the PulS homologue OutS containing the binding peptide of the S-domain of the PulD homologue OutD^35^ (Fig. 3a). OutS has the same structural fold as PulS and functionally interacts in a similar way with OutD as PulS does with PulD^36^,^37^. In fact, OutS can substitute for PulS^30^.

Of the residues lining the PulS binding cleft, Q38 on α-helix 1 and Q95 on α-helix 3 appeared to be good candidates (in distance and orientation relative to the PulD peptide) for substitution by cysteines: Q38C might cross-link with two residues of the PulD S-domain, A649C and F654C, while Q95C might cross-link with A643C of the PulD S-domain (Fig. 3a). All of the pairs tested produced PulD^fl^ multimers, albeit less efficiently than observed with wild-type PulD^fl^ and wild-type PulS (Fig. 3b). An upper shift in the electrophoretic migration of PulD^fl^_A643C_ monomers recognized by anti-PulD antibodies indicated that it cross-linked with high efficiency to PulS_Q95C_, while PulD^fl^_A649C_ and PulD^fl^_F654C_ showed no or limited cross-linking with PulS_Q38C_, respectively (Fig. 3b). PulD^fl^_A643C_ monomers and multimers were also recognised efficiently by anti-PulS antibodies (Fig. 3b). Cross-linking was specific between the engineered cysteines A643C and Q95C, as efficient cross-linking was not achieved when either of the variants was produced in the presence of the wild-type binding partner (Fig. 3c). The slower migrating cross-linked PulD^fl^_A643C_-PulS_Q95C_ hetero-dimer disappeared upon treatment with dithiothreitol (DTT), which resulted in a concomitant increase in the amount of non-cross-linked PulD^fl^_A643C_ monomers. In addition, broadening of the multimer band upon DTT treatment also suggested that PulD^fl^_A643C_ multimers were cross-linked to PulS_Q95C_ (Fig. 3c). Phenol treatment dissociated the multimers and predominantly resulted mainly in an increase in the amount of cross-linked PulD^fl^_A643C_-PulS_Q95C_ hetero-dimers (Fig. 3c). Neither phenomenon occurred with any of the other combinations or between wild-type proteins and the variants (Fig. 3c).

**Figure 4:**
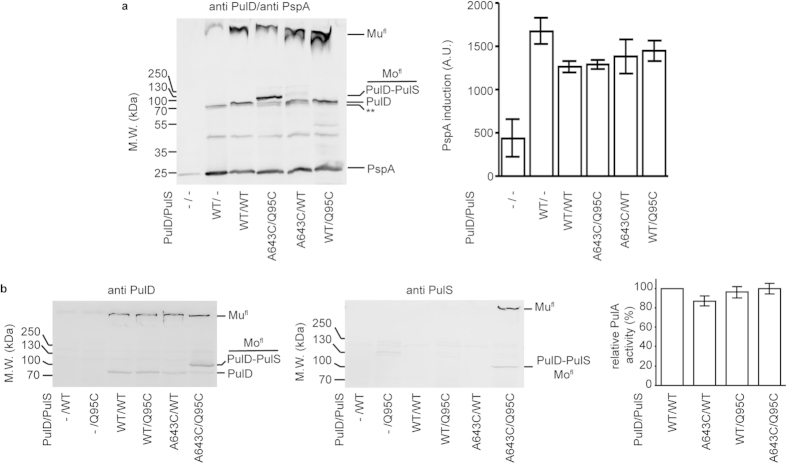
Effects on the functional properties of PulD^fl^ following cysteine bridge formation between PulD^fl^ and PulS *in vivo*. (**a**) Analysis of the induced PspA response upon PulD^fl^ multimer production. (**b**) PulA activity upon its secretion by the type II secretion system in the presence of WT or cysteine variants of PulD^fl^ and PulS, as indicated. The percentage PulA activity was calculated relative to that obtained in the presence of WT PulD^fl^ and PulS. Immunoblots after SDS-PAGE of total cell extracts were stained with anti PulD, anti PulS and anti PspA antibodies as indicated. Errors represent S.D. over 3 independent measurements. Mu^fl^ and Mo^fl^ indicate the migration position of multimeric and monomeric PulD^fl^ species, respectively. ** Indicates trimmed PulD^fl^ monomers that escape outer membrane targeting^20^.

Having established that at least the majority of the PulD^fl^_A643C_ multimers formed were cross-linked to PulS_Q95C_, we next investigated whether these multimers behaved like the wild-type upon production in *E. coli*; i.e., do they induce the Psp response and permit PulA secretion. A Psp response is induced when PulD multimers associate with or insert into the inner membrane. For example, PspA production is high when even a small amount of PulD^fl^ multimers is assembled in the absence of PulS (Fig. 4a). In this case, PulD^fl^ is no longer transported to the outer membrane, which results both in rapid PulD^fl^ degradation and in insertion of PulD that escapes degradation into the inner membrane, leading to PspA induction. PspA induction is lower, but still above background, when PulD^fl^ is rendered resistant to degradation and is targeted to the outer membrane by PulS (e.g., Fig. 4a). PspA induction remained at similar levels when either PulD^fl^_A643C_ or PulS_Q95C_ were produced in the presence of their wild-type binding partner or when PulD^fl^_A643C_ and PulS_Q95C_ were produced together and cross-linked (Fig. 4a). Thus, cross-linked hetero-dimers were likely transported to the outer membrane. Furthermore, since PspA levels were above background, a small proportion of the PulD^fl^_A643C_ multimers apparently inserted into the inner membrane and formed small pores, as is the case with wild-type PulD and PulS^21^,^22^.

PulS production and lipidation are critical to achieve PulA secretion through PulD^30^,^38^. Regardless of the presence of the substituted cysteines and regardless of the formation of a disulfide bridge, all multimeric secretins secreted PulA efficiently (Fig. 4b). Hence, it appears that PulD^fl^_A643C_-PulS_Q95_ cross-linked secretins are fully functional and that PulS dissociation is not required to allow PulD assembly and function.

### Final PulD^28–42/259–660^ folding steps are efficient in phosphatidylethanolamine, but not in phosphatidylglycerol

**Figure 5:**
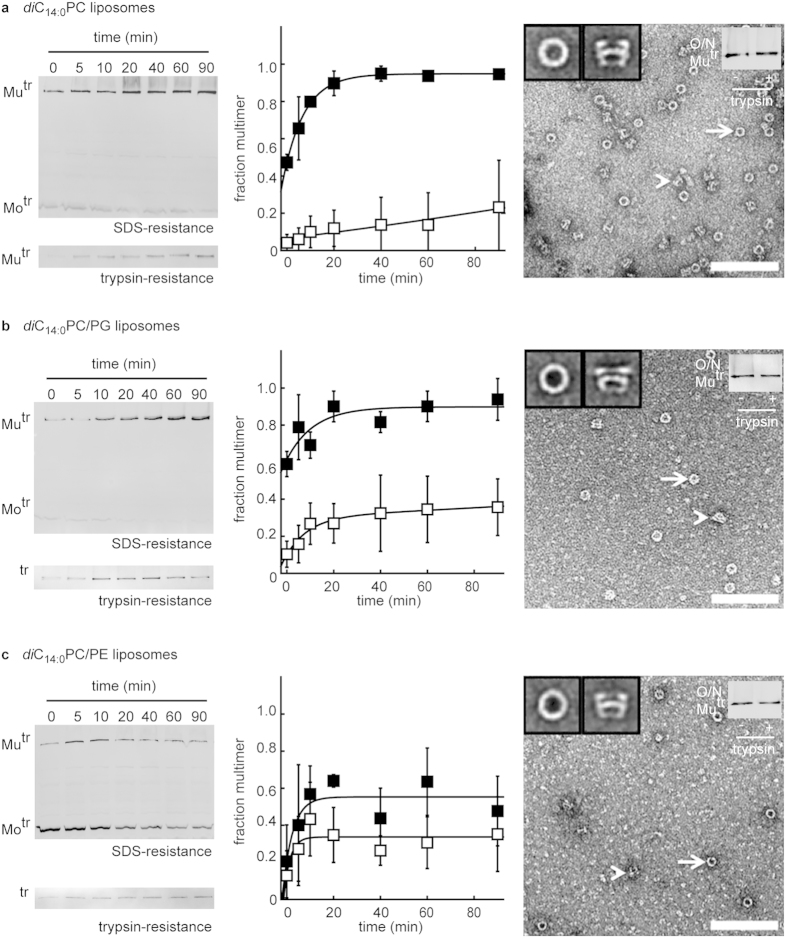
Kinetics of PulD^28–42/259–660^ folding in C_14:0_-liposomes containing heterogeneity in the lipid headgroups. (**a**) Multimerisation kinetics by acquisition of SDS- (◼) and trypsin- (◻) resistance in the presence of 53 mM *di*C_14:0_-phosphatidylcholine (PC) liposomes. (**b**) Multimerisation kinetics by acquisition of SDS- (◼) and trypsin- (◻) resistance in the presence of 53 mM *di*C_14:0_-phosphatidylcholine/glycerol (PC/PG) liposomes (60:40 mol/mol). (**c**) Multimerisation kinetics by acquisition of SDS- (◼) and trypsin- (◻) resistance in the presence of 53 mM *di*C_14:0_-phosphatidylcholine/ethanolamine (PC/PE) liposomes (90/10 mol/mol). Errors represent S.D. over 3 independent measurements. (**a**–**c**) also show trypsin-resistance after overnight (O/N) incubation in the respective liposomes. Mu^tr^ and Mo^tr^ indicate the migration position of multimeric and monomeric PulD^28–42/259–660^ species, respectively. (**a**–**c**) include field images obtained by transmission electron microscopy of negatively stained PulD^28–42/259–660^ multimers solubilised from the respective liposomes in dodecylmaltoside (scale bar is 100 nm). The arrow and arrowhead show the top and side view, respectively. The insets show the average images of top (left) and side (right) view.

The fast acquisition of the trypsin-resistant state in the presence of *E. coli*-lipids, compared to that in the presence of lecithin, demonstrated the importance of the lipid composition for efficient PulD^28–42/259–660^ folding. To explore this observation further, PulD^28–42/259–660^ was produced in the presence of a series of liposomes with different headgroup compositions and hydrophobic thicknesses. Whether PulD^28–42/259–660^ could form native multimers in these liposomes was tested by electron microscopy (EM) of PulD^28–42/259–660^ solubilised from the liposomes by dodecylmaltoside. These procedures provide well-established, standard criteria for determining the extent of PulD^28–42/259–660^ folding and assembly^15^,^25^,^39^. Data from a series of comparisons are shown in Fig. 5. PulD^28–42/259–660^ appears as two stacked rings when examined by EM^15^. As previously, particle averaging revealed two orientations: a stack when viewed in the plane of the membrane (side view) and a disc perpendicular to the axis of symmetry (top view) (Fig. 5).

A first important difference between lecithin- and *E. coli*-liposomes is the composition of the lipid headgroups. To investigate whether the headgroup-composition of the membrane influences PulD^28–42/259–660^ folding into its native state, PulD^28–42/259–660^ folding was initiated in synthetic liposomes prepared from phospholipids with acyl chains containing 14 carbons. Bilayers containing C_14_-lipids are close in hydrophobic thickness to that of the outer membrane^40^. To assess the role of the lipid headgroup composition, 10% *di*C_14:0_PE or 40 % *di*C_14:0_PG were incorporated into the *di*C_14:0_PC-bilayer. Whereas the PG-fraction used in the *di*C_14:0_PC-liposomes might be considered rather large, the mole-fraction of the PE-lipid is much lower than it is in bacterial membranes. For example, phospholipids in *E. coli* K-12 outer membranes have at least 75 % PE-headgroups and at the most 25 % PG-headgroups^41^. However, the PE-fraction used is the highest that allows one to probe the importance of the PE-headgroup properties whilst maintaining membrane fluidity at the PulD^28–42/259–660^ synthesis temperature (30 °C).

PulD^28–42/259–660^ multimerised with high efficiency after 90 min into *di*C_14:0_PC- and *di*C_14:0_PC/PG-liposomes (90 ± 10% and 94 ± 2%, respectively; Fig. 5a,b). Multimerisation was much less efficient in *di*C_14:0_PC/PE-liposomes (up to 55 ± 13% on average; Fig. 5c), presumably due to the proximity of the phase transition temperature in the presence of *di*C_14:0_PE^42^,^43^. However, whereas at most approximately a third of PulD^28–42/259–660^ multimers acquired trypsin-resistance within 90 min in the presence of *di*C_14:0_PC- and *di*C_14:0_PC/PG-liposomes (16 ± 5% and 35 ± 4% of total PulD, respectively, Fig. 5a,b), more than half of the multimerised PulD^28–42/259–660^ was trypsin-resistant after 20 min in *di*C_14:0_PC/PE-liposomes (35 ± 10% of total PulD, Fig. 5c). Although the amount of trypsin-resistant PulD^28–42/259–660^ multimers in *di*C_14:0_PC- and *di*C_14:0_PC/PG-liposomes was modest after 90 min, a significantly higher amount acquired trypsin-resistance after overnight incubation, indicating that the high trypsin-sensitivity measured at shorter times was not due to the physicochemical properties of the lipid composition used or to saturation of the liposomes with inserted PulD^28–42/259–660^ multimers (Fig. 5a,b). EM confirmed that PulD^28–42/259–660^ multimers attained their native structure in all of the membranes used (Fig. 5a–c). Thus, although the yield of PulD^28–42/259–660^ multimers was lower in the presence of *di*C_14:0_PC/PE-liposomes, the data suggest that late stages in PulD^28–42/259–660^ assembly occur more rapidly into *di*C_14:0_PC/PE-liposomes than into *di*C_14:0_PC- and *di*C_14:0_PC/PG-liposomes.

**Figure 6:**
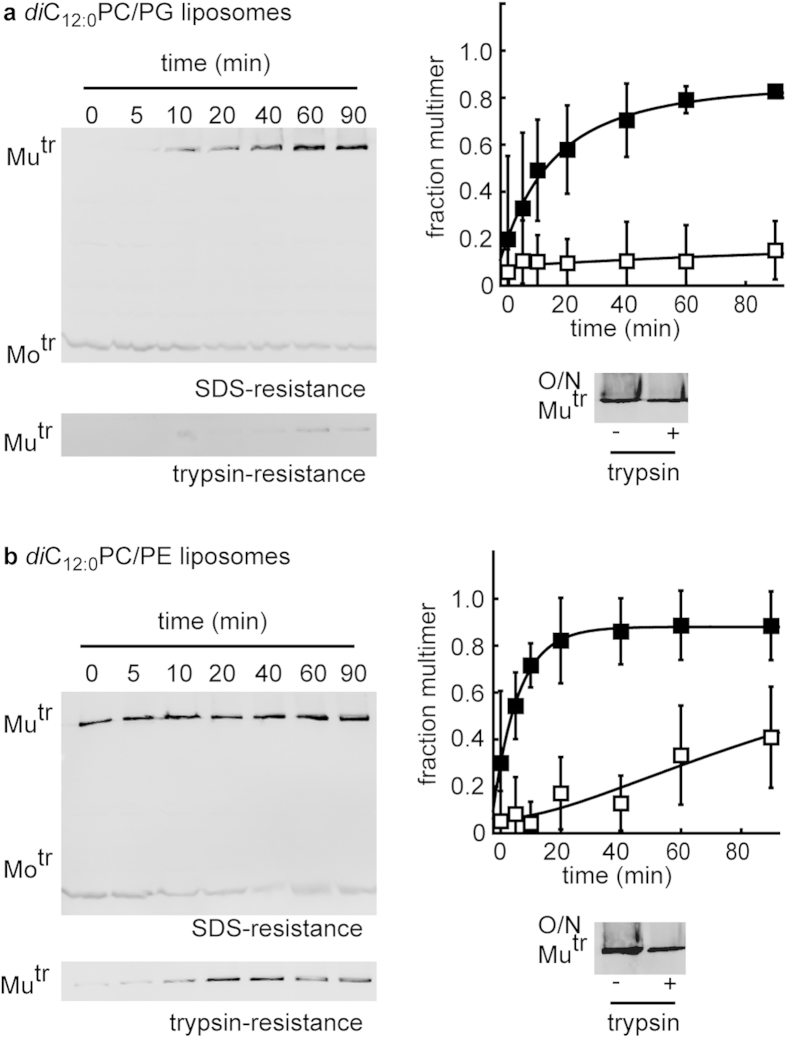
Kinetics of PulD^28–42/259–660^ folding in C_12:0_-liposomes containing heterogeneity in the lipid headgroups. (**a**) Multimerisation kinetics by acquisition of SDS- (◼) and trypsin- (◻) resistance in the presence of 53 mM *di*C_12:0_-phosphatidylcholine (PC/PG) liposomes (60/40 mol/mol). (**b**) Multimerisation kinetics by acquisition of SDS- (◼) and trypsin- (◻) resistance in the presence of 53 mM *di*C_12:0_-phosphatidylcholine/ethanolamine (PC/PE) liposomes (90/10 mol/mol). Errors represent S.D. over 3 independent measurements. (**a**,**b**) also show trypsin-resistance after overnight (O/N) incubation in the respective liposomes. Mu^tr^ and Mo^tr^ indicate the migration position of multimeric and monomeric PulD^28–42/259–660^ species, respectively.

Because of the low yield of the PulD^28–42/259–660^ multimers in *di*C_14:0_PC/PE-liposomes, PulD^28–42/259–660^ assembly into liposomes containing 60 % *di*C_12:0_PC and 40 % *di*C_12:0_PG was compared with that in liposomes containing 90 % *di*C_12:0_PC and 10 % *di*C_12:0_PE. The latter liposomes remain more fluid than *di*C_14:0_PC/PE-liposomes with the same headgroup ratio and should produce a higher yield of PulD^28–42/259–660^ multimers. After 90 min PulD^28–42/259–660^ multimerised with high yields in the presence of both types of liposomes (87 ± 3% and 89 ± 1% in *di*C_12:0_PC/PG-liposomes and *di*C_12:0_PC/PE-liposomes, respectively (Fig. 6)). However, multimerisation was markedly slower in *di*C_12:0_PC/PG-liposomes at 0.049 ± 0.009 min^−1^ compared to 0.115 ± 0.008 min^−1^ in *di*C_12:0_PC/PE-liposomes (Fig. 6). Trypsin resistance was consistently higher after 20–60 min in *di*C_12:0_PC/PE-liposomes, while it remained low throughout the first 90 min in in *di*C_12:0_PC/PG-liposomes (Fig. 6). Overnight incubation allowed the acquisition of higher trypsin resistance in *di*C_12:0_PC/PG and *di*C_12:0_PC/PE-liposomes (Fig. 6).

Together, the data indicate that PulD^28–42/259–660^ folding was accelerated in *di*C_12:0_PC and *di*C_14:0_PC-liposomes by including lipids with PE-headgroups.

### Thick bilayers accelerate final PulD^28–42/259–660^ folding steps

**Figure 7:**
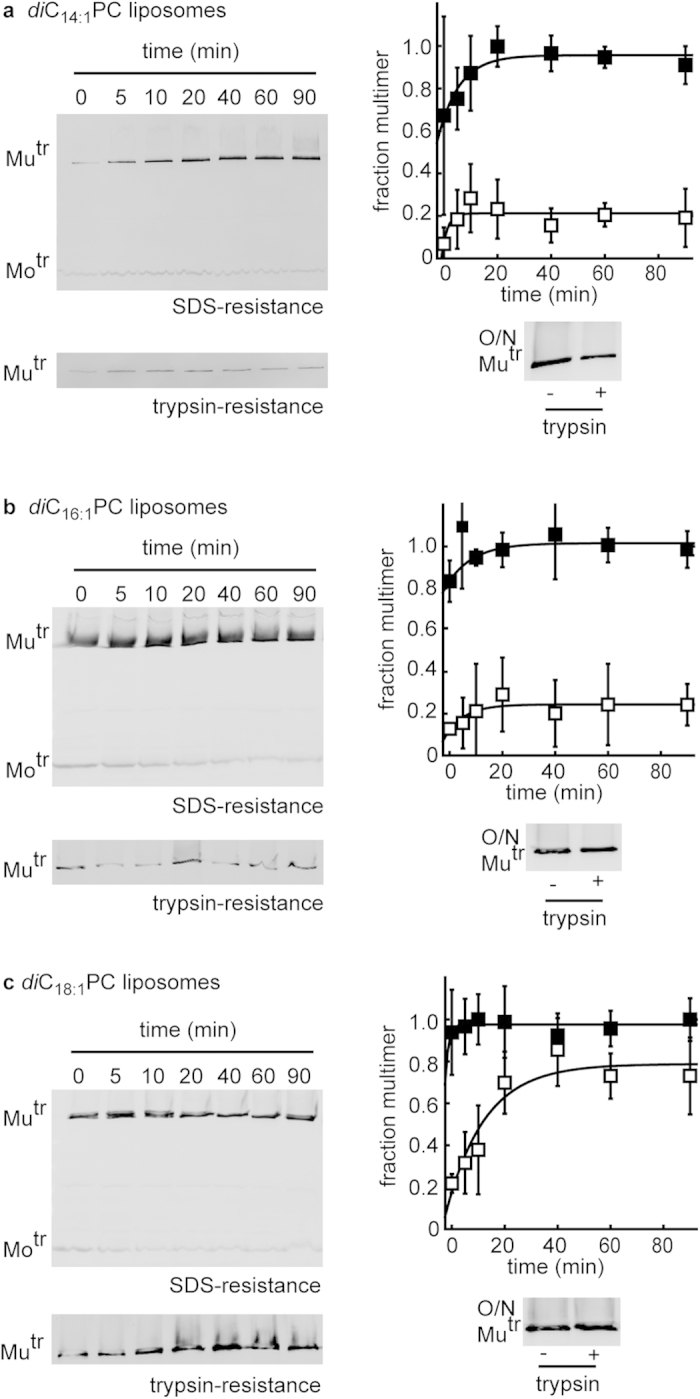
Kinetics of PulD^28–42/259–660^ folding in phosphatidylcholine (PC) liposomes with increasing hydrophobic thickness. (**a**) Multimerisation kinetics by acquisition of SDS- (◼) and trypsin- (◻) resistance in the presence of 53 mM *di*C_14:1_PC-liposomes. (**b**) Multimerisation kinetics by acquisition of SDS- (◼) and trypsin- (◻) resistance in the presence of 53 mM *di*C_16:1_PC-liposomes. (**c**) Multimerisation kinetics by acquisition of SDS- (◼) and trypsin- (◻) resistance in the presence of 53 mM *di*C_18:1_PC-liposomes. Errors represent S.D. over 3 independent measurements. (**a–c**) also show trypsin-resistance after overnight (O/N) incubation in the respective liposomes. Mu^tr^ and Mo^tr^ indicate the migration position of multimeric and monomeric PulD^28–42/259–660^ species, respectively.

Besides the differences in lipid headgroup composition between the *E. coli* and lecithin-liposomes, the lecithin bilayer has a higher hydrophobic thickness than *E. coli*-bilayers. *E. coli* lipids predominantly contain acyl chains that are 16 carbons in length^44^, but soy bean lecithin primarily contains lipids with 18 carbon long acyl-chains^45^. Considering that the hydrophobic thickness of the outer membrane is slightly less than that of bilayers of *E. coli* phospholipids (the acyl chains on LPS molecules usually have 14 carbons^1^,^40^) the increased bilayer thickness of lecithin might delay the acquisition of PulD^28–42/259–660^ trypsin-resistance. The folding of PulD^28–42/259–660^ was therefore measured in synthetic liposomes containing only *di*C_16:1_PC or *di*C_18:1_PC lipids, as well as in *di*C_14:1_PC-liposomes, all of which remain fluid under the experimental conditions used. PulD^28–42/259–660^ SDS-resistance was acquired quickly in the presence of *di*C_14:1_PC-, *di*C_16:1_PC- and *di*C_18:1_PC-liposomes with rates of 0.125 ± 0.049, 0.103 ± 0.051 and 0.189 ± 0.110 min^−1^, respectively and with efficiencies of 97 ± 7%, 101 ± 5% and 98 ± 5% after 90 min (Fig. 7a–c). However, whereas only 21 ± 5% and 23 ± 6% was trypsin-resistant after 90 min in the presence of *di*C_14:1_PC- and *di*C_16:1_PC-liposomes, respectively (Fig. 7a,b), the majority of the multimerised PulD^28–42/259–660^ (72 ± 18 %) adopted the trypsin resistant state gradually with increasing multimerisation in *di*C_18:1_PC-liposomes (Fig. 7c). Higher levels of trypsin-resistance were also achieved in the presence of *di*C_14:1_PC- and *di*C_16:1_PC-liposomes after overnight incubation (Fig. 7a,b). Thus, although the final stages of PulD^28–42/259–660^ folding were slow in thick lecithin-liposomes, PulD^28–42/259–660^ folding occurred rapidly in the presence of pure *di*C_18:1_PC-liposomes. In contrast, while PulD^28–42/259–660^ folding was favoured in thinner *E. coli*-membranes, folding was slow in pure *di*C_16:1_PC-liposomes.

The results suggest that the hydrophobic thickness of the membrane affects the rate of PulD^28–42/259–660^ folding; however, hydrophobic thickness alone is not a critical determinant in PulD^28–42/259–660^ folding.

## Discussion

This report examines the factors required for the folding and assembly of the OMP PulD, whose biogenesis is independent of the general OMP-specific assembly machinery (BAM)^23^. Previous *in vivo* and *in vitro* studies^20^,^21^,^46^ demonstrated that the lipoprotein PulS plays an essential role in delivering PulD to the outer membrane. Here we show that PulS can improve the efficiency of early PulD multimerisation steps *in vitro*, but we failed to observe any major influence on later steps in PulD folding corresponding to the transition of the prepore into the native structure^29^. Nonetheless, PulS does not have to dissociate from its substrate for PulD to complete this transition. These observations clearly show that the PulD assembly pathway is quite different from the general pathway used by most OMPs, in which the broad specificity chaperones SurA and Skp must release their substrates near the membrane surface, passing them on to BAM for assisted membrane insertion^2^. Other members of the PulS family likely play equivalent roles in secretin assembly^30^,^37^. Dedicated chaperones AspS and PilF to the secretins GspD from *Vibrio* and *E. coli* EPEC and PilQ from *P. aeruginosa*, respectively, catalyse Bam-independent secretin assembly^9^,^10^,^47^,^48^, reflecting even closer involvement of this chaperone in secretin assembly than is the case for PulS. Other dedicated chaperones have somewhat different roles. In *Neisseria meningitidis*, for example, the chaperone PilW is critical for secretin PilQ stability^49^, while PilQ assembly is reported to rely on BAM^8^. The *Pseudomonas aeruginosa* secretin HxcQ is a lipoprotein that uses the Lol-pathway to reach the outer membrane^50^ and might be stabilised by a second, smaller protein^47^. It is unknown whether HxcQ assembly is BAM-dependent. A systematic study of secretin assembly and folding pathways by a combination of *in vivo* and *in vitro* approaches would further define these differences and reveal whether secretins can be divided into two biogenesis classes: one relying totally on classical BAM-mediated membrane insertion and for which dedicated chaperones have roles not directly related to assembly, and a second class independent of BAM for membrane insertion, but for which dedicated chaperones are required for the correct localisation and for the catalysis of assembly steps of at least some.

Since PulS only catalyses initial PulD assembly steps, the question remains how PulD inserts into the membrane. Membrane insertion could depend on an as yet-unidentified assembly machinery, although this seems unlikely in view of its ability to insert both into artificial, protein-free liposomes *in vitro* and into the *E. coli* inner membrane when PulS is absent *in vivo*^21^,^22^. However, we observe that the lipid composition of the membrane influences PulD folding *in vitro* in an unusual fashion. Neither the lipid headgroup composition nor the hydrophobic thickness themselves appeared to be critical for folding. If the headgroup composition were critical, then PulD^28–42/259–660^ folding would have been slow in *di*C_18:1_PC-liposomes, whereas the contrary was observed. If membrane thickness were the critical factor, then PulD^28–42/259–660^ folding would have been more efficient in lecithin compared to *E. coli* lipid, whereas the contrary was observed. Instead, we propose that general physical membrane properties, like membrane curvature and membrane-stored energy, drive efficient PulD insertion, both *in vitro* and *in vivo*. Stored energy is high in PE-containing bilayers (as in *E. coli* extract liposomes) because of the non-bilayer packing conformations of the PE-lipids that lead to an increase in curvature stress^51^,^52^, and also in thick membranes composed of lipids with long saturated or mono-unsaturated acyl chains (as in *di*C_18:1_PC)^53^. In contrast, thick lecithin liposomes, which contain a high number of poly-unsaturated acyl chains, and thin PC-/PG-liposomes form highly elastic membranes with little stored energy.

As PulD insertion appears to be tuned to suit the *in vivo* membrane composition, the observations reported here might rationalise why PulD assembly is Bam-independent. BAM comprises five proteins: four peripheral lipoproteins (BamB-E) and one membrane embedded protein, BamA, which forms the central component that catalyses OMP insertion^3^. High-resolution structures and simulations reveal how the 16-stranded β-barrel of BamA distorts the membrane around strands 1 and 16, providing an access route for OMP insertion into the membrane^6^ by lowering the energy barrier for OMP membrane insertion in the presence of PE-containing phospholipids^5^,^7^. If PulD exploits membrane-stored energy for its membrane insertion, it would not need a system such as BAM that lowers the energy. Insertion in this manner likely requires a high level of organisation to measure the amount of energy stored in the membrane, for example by sensing lateral pressure. Formation of the PulD prepore^39^ to organise the C-domains could provide a means to achieve this. Therefore, the stability of the prepore structure could be a critical parameter in determining the fate of assembling PulD secretins.

How general is this phenomenon of BAM-independent OMP assembly? Although BAM-independent assembly was initially reported for PulD^23^ and then for other secretins in the same family^10^, the OMPs CsgG, GfcC and Wza were also shown recently to exhibit Bam-independent assembly. Like PulD, they also appear to form prepore structures^9^,^54^,^55^. Like PulD^29^,^39^, all of these complexes require the coalescence of multiple subunits to form a single transmembrane pore or channel. We hypothesise that a common assembly mechanism based on achieving a critical stability in the prepore and membrane-assisted insertion represents a new paradigm for complex OMP assembly. The characteristics that determine whether OMP assembly is BAM-dependent or not might be encoded in the three-dimensional structure of the OMP, which remains to be determined at high-resolution for secretins. *In vitro* analysis of the folding of OMPs with diverse structural features in the presence and the absence of BAM would greatly advance our understanding of the mechanisms involved.

## Methods

### Strains, plasmids, cloning and site-directed mutagenesis

Cloning and PulD^fl^ functional assays were performed in *E. coli* K-12 Pap105 (∆(*lac-pro*) F’ (*lacI*^*q1*^ ∆*lacZM15 proAB*^+^ Tn*10*)). Cells were grown at 30 °C in Luria Bertani medium supplemented with ampicillin (100 μg/ml) and chloramphenicol (25 μg/ml) as appropriate.

Plasmids encoding for PulD variants were obtained by site-directed mutagenesis on the plasmids pCHAP3635^56^ and pCHAP362^57^. The first is a pSU18 derivative that allows high levels of PulD production for cross-linking and PspA response assays, whilst the second is a pHSG575 derivative for a low production level used in secretion assays. PulS variants were generated from a pUC19 derived vector containing the *pulS* gene (pCHAP585)^56^. Primers used for mutagenesis are listed in Table 1.

To produce PulS_Q95C_ in the presence of all other Pul proteins, the *pulS* gene was mutagenised through a cloning sequence rather than by site-directed mutagenesis. This was required because of the large size of the plasmids carrying the entire *pul* operon. First, the DNA fragment encoding for all the Pul proteins (except PulD^fl^) was amplified from pCHAP1226^58^ and ligated into pCHAP231^38^ using restriction sites PsiI and HindIII. This created the plasmid pCHAP3402 that encodes for all the Pul proteins except PulD and has unique AscI and AsiSI restriction sites flanking a 2929 bp fragment carrying the *pulS* gene. Two separate, partially overlapping amplicons were generated to cover the entire 2929 bp: one fragment from the AsiSI-site up to the codon for Q102 on the *pulS* gene and a second from the codon for S89 on the *pulS* gene to the AscI-site. Primers annealing to the *pulS* gene carried the required codon change to substitute amino acid Q95 into C in PulS (Table 1, primers ING339 and 340). Primers annealing near the AsiSI and AscI-sites (in italics) are 5′-AAACGACGGCCAGTGAATTCAG*GCGATC*GCCGTTGAAGGTC-3′ and 5′-GACCATGATTACGCCAAGCTTTAAC*GGCGCGCC*TGGCGG-3′, respectively. Both primers also contained an EcoRI and a HindIII-site (underlined), respectively, to enable an intermediate cloning step into the pUC19-vector amplified with the primers 5′-GAATTCACTGGCCGTCGTTTTAC-3′ and 5′-AAGCTTGGCGTAATCATGGTC-3′. The three fragments were assembled using the Gibson assembly master mix (NEB) to give plasmid pCHAP3404. The fragment carrying the mutagenised *pulS* gene was excised from pCHAP3404 using AsiSI and AscI and was ligated into the plasmid pCHAP3402 digested with the same enzymes to give pCHAP3405. All constructs were verified by DNA sequencing.

### Analysis of PulD^fl^-PulS cross-linking efficiency

Cells were transformed with the appropriate combination of two plasmids, one encoding for wild-type PulD^fl^ or a single cysteine variant of PulD^fl^ (A643C, A649C or F654C) and one for wild-type PulS or a single cysteine variant of PulS (Q38C or Q95C). The cells of 1 ml of the overnight culture were collected and resuspended in SDS sample buffer (4% SDS, 62.5 mM Tris (pH 6.8), 20% glycerol) supplemented with 10 mM dithiotreitol (DTT), as indicated, to a density of 10 D_600nm_/ml. Where indicated, PulD multimers were dissociated by phenol extraction and dissolved at the same concentration in SDS sample buffer with or without DTT, as indicated. All samples were boiled and 0.05 D_600nm_/ml of each was loaded. Proteins were separated on 10% or 15% polyacrylamide (37.5:1 acrylamide/bisacrylamide) gels or gels composed of stacked layers of 10% and 15% and analysed by immunoblotting with antibodies against PspA, PulS, PulD and OmpF, as indicated. Bands were analysed by densitometry.

### PspA induction and PulA secretion assay

PspA induction was measured from the same cells used in the cross-linking assays. An empty vector and cells producing wild-type PulD in the absence of PulS were used as negative and positive controls, respectively.

PulA activity following its secretion to the outer surface was measured upon induction of the entire *pul* operon with 0.4% maltose in cells transformed with one of two plasmids encoding for the entire set of Pul proteins except PulD^fl^, pCHAP3402 (for wild-type PulS) or pCHAP3405 (for PulS_Q95C_), and either pHSG575 (empty vector), pCHAP362 (wild-type PulD^fl^) or pCHAP3406 (PulD^fl^_A643C_). Pullulanase secretion was measured as a fraction of the pullulanase enzymatic activity on the bacterial surface in whole cells compared to that of octyl-polyoxyethylene lysed bacteria and relative to the activity upon PulA secretion in the presence of wild-type PulS and PulD^fl ^56,59.

### Liposome preparation

Appropriate amounts of lecithin (Sigma), *E. coli* polar extract, *di*C_12:0_PC, *di*C_12:0_PE, *di*C_14:0_PC, *di*C_14:0_PG, *di*C_14:0_PE, *di*C_14:1_PC, *di*C_16:1_PC or *di*C_18:1_PC (Avanti Polar Lipids) in solvent (as supplied) were dried under a gentle stream of nitrogen followed by evaporation of residual chloroform under vacuum. Dried lipids were hydrated to 20–200 mg/ml (as appropriate), vortexed and sonicated for 15 min in a water bath.

### sPulS production and purification

Production and purification of sPulS is described elsewhere^19^. Briefly, cells containing the plasmid for the expression of MalE-PulS with an N-terminal hexahistidine-tag were grown to a D_600_ = 0.5 and induced with 0.5 mM IPTG for 4 h. Cells were harvested, lysed and debris was removed by centrifugation. The supernatant was applied to a nickel charged HiTrap column for affinity purification. After elution, MalE-PulS containing fractions were dialysed and digested overnight with Factor Xa. sPulS was further purified by cation exchange (HiTrap SP-Sepharose column) and gel filtration (HiLoad 16/60 Superdex 200 column).

### PulD synthesis

PulD was synthesised by *in vitro* translation using an RTS100 *E coli* kit (5 Prime) as described^25^,^29^ in the presence of 10 ng DNA (pCHAP3731 (PulD^fl^), pCHAP3716 (PulD^28–42/259–660^), pCHAP3803 (PulDΔS^28–42/259–598^), 10 to 60 μg liposomes and 0.2 μg sPulS (as indicated) per μl RTS100 at 30 °C. Although the RTS100 kits were centrifuged at 100000 *g* for 30 min before use to remove most of the *E. coli* membranes, the trace amounts that remain are sufficient to allow limited PulD assembly. Synthesis was arrested with 3 ng streptomycin per μl of reaction after 6 min for initial multimerisation experiments, after 10 min in all other kinetic experiments and for at least 6 h for structural characterisation. Synthesis reactions were further incubated for at least 6 h at 30 °C for complete folding to occur. Mixed multimers were produced by priming the reaction with the relevant DNAs in a 1:1 ratio. PulDΔS^28–42/259–598^ was used as a control for the effects of the addition of sPulS to the reaction mixture. PulDΔS^28–42/259–598^ no longer has the S-domain that binds PulS and behaves in all experiments performed as PulD^28–42/259–660^ in the absence of sPulS. Monomeric and multimeric PulD were separated in SDS on a 10 % polyacrylamide (37.5:1 acrylamide/bis-acrylamide) gel without heating to 100 °C, transferred to nitrocellulose and analysed by immunoblotting with an antibody raised against native PulD-multimers. Bands corresponding to multimeric and monomeric PulD were analysed by densitometry. Resulting transients were fitted to a single exponential equation using Kaleidagraph 4.0. Fitting parameters are reported in the text.

### Folding kinetics followed by SDS treatment

Folding transients of PulD^28–42/259–660^ were obtained by mixing aliquots of the synthesis reaction at the time points indicated with SDS sample buffer in a 1:1 ratio to arrest folding and incubated on ice for 1 h before analysis by SDS-PAGE.

### Folding kinetics followed by limited proteolysis by trypsin digestion

Trypsin was added to PulD^28–42/259–660^ aliquots at the times indicated to a final concentration of 4 μg/μl and incubated on ice for 5 min. Reactions were blocked using 150 ng/ml Pefabloc (Interchim) before mixing with SDS sample buffer for analysis. The fraction of trypsin resistant multimers was determined relative to the amount of SDS-resistant multimer at the endpoint of the reaction.

### Protein solubilisation and transmission electron microscopy (TEM)

Liposomes, purified as above, were resuspended in 100 mM Tris, pH 7.5, and 500 mM NaCl and diluted twice in 2% DDM. The lipid to detergent ratio was typically 1:5 (w/w) for solubilisation. For negative staining, 4 μl of sample was adsorbed onto carbon film-coated copper EM grids, washed with three droplets of pure water and subsequently negative stained with 2% (w/v) uranyl-acetate. The prepared grids were imaged using a Philips CM10 TEM (FEI, Eindhoven, The Netherlands) operating at 80 keV. Images were recorded on a side-mounted Veleta 2 K × 2 K CCD camera (Olympus, Germany) at a magnification of 130000. The pixel size at the sample level is 3.7 Å. Image processing was performed in the EMAN2 software package^60^. The images were contrast transfer function corrected and the particles were semi-automatically selected. e2refine2d was used to classify the particles. This program produces reference-free class averages from a population of mixed, unaligned particle images. The representative class average with the best signal-to-noise ratio were selected and gathered in a gallery.

## Additional Information

**How to cite this article**: Huysmans, G. H. M. *et al.* Lipids assist the membrane insertion of a BAM-independent outer membrane protein. *Sci. Rep.* **5**, 15068; doi: 10.1038/srep15068 (2015).

## Figures and Tables

**Table 1:** Mutagenesis primers and plasmid numbers used in this study.

Name	DNA sequence (5′to 3′)	substitution	plasmid pCHAP
ING329	CCGCGCCAGGATACCGCC*TGT*TTCCGTCAGGTCAGCGCC	PulDA643C	3363/3406
ING330	GGCGCTGACCTGACGGAA*ACA*GGCGGTATCCTGGCGCGG		
ING333	CGCGTTCCGTCAGGTCAGC*TGC*GCTATCGACGCGTTCAATC	PulDA649C	3365
ING334	GATTGAACGCGTCGATAGCG*CAG*CTGACCTGACGGAACGCG		
ING337	CAGCGCCGCTATCGACGCGT*GCA*ATCTGGGAGGCAATCTAT	PulDF654C	3367
ING338	ATAGATTGCCTCCCAGATT*GCA*CGCGTCGATAGCGGCGCTG		
ING339	CAGCGCGCAGCTATATCAA*TGT*CTCCAGCAGGACAGTACCC	PulSQ95C	3376/3404/3405
ING340	GGGTACTGTCCTGCTGGAG*ACA*TTGATATAGCTGCGCGCTG		
ING345	GTCAGGCCCAGTTAGAG*TGT*CTTGCCTCCGTCGCCGC	PulSQ38C	3377
ING346	GCGGCGACGGAGGCAAG*ACA*CTCTAACTGGGCCTGAC		

The codon change is highlighted in italic letters.
